# A Robust Functional Genomics Approach to Identify Effector Genes Required for Thrips (*Frankliniella occidentalis*) Reproductive Performance on Tomato Leaf Discs

**DOI:** 10.3389/fpls.2018.01852

**Published:** 2018-12-13

**Authors:** Ahmed M. Abd-El-Haliem, Suzanne W. Hoogstrate, Robert C. Schuurink

**Affiliations:** Department of Plant Physiology, Swammerdam Institute for Life Sciences, University of Amsterdam, Amsterdam, Netherlands

**Keywords:** thrips bioassay, resistance, effectors, functional-genomics, tomato

## Abstract

Thrips (*Frankliniella occidentalis*) is a persistent plant pest that is able to vector pathogenic viruses. Natural plant resistance to thrips has become a prominent breeding target in commercial crops. The main reason for this is the shift toward banning key pesticides used for controlling thrips infestations and the lack of effective alternatives. Despite this urgent need for crop plants that are resistant, or tolerant, to thrips infestation, the toolbox for studying genetic resistance to this insect is still underdeveloped. Essentially, there is a lack of robust protocols for the screening and identification of thrips genes relevant to its performance on crop plants. To bridge this gap, we have developed a functional analysis screening method. Our approach relies on the, *Agrobacterium tumefaciens*-mediated, homogeneous, and transient ectopic expression of thrips genes in large tomato leaf discs followed by the assessment of thrips reproductive performance. The setup is designed to maintain gene expression during the course of the assay, where GFP signal in the control treatment is used as a reporter of expression. The screen is conducted in a climate box under controlled settings. As a result, multiple genes can be screened for their effect on thrips reproductive performance in a single experiment and in a relatively small space, without the need for generating stable transgenic plants. The method also eliminates the need for a greenhouse equipped to accommodate the combination of *A. tumefaciens*-infiltrations and thrips infestations. It is not only flexible and convenient for screening genes encoding putative thrips effectors but also for plant resistance genes or effector-targets of host plants and can be adapted for other crop plants, or other herbivorous arthropods.

## Introduction

Western flower thrips, *Frankliniella occidentalis* (Pergande), is a piercing-sucking pest which puncture plant cells with a specialized mandible and then ingest the cell content (Hunter and Ullman, 1989, 1992). Damaged cells die and form a phenotype visible on the surface of plant leaves and fruits, commonly termed as “silvery damage.” If the damaged cells are in developing organs like young leaves, small fruits or flower pods, then infestation usually results in the deformation of these organs. On top of this, thrips is a successful vector in transmitting several economically important plant viruses, like the tomato spotted wilt virus (TSWV), from infected to healthy plants (Steenbergen et al., 2018). The search for genetic resistance against thrips has been ongoing for more than two decades (Fery and Schalk, 1991; Douglas, 2018). An increasingly used strategy to breed resistant plants or to understand the arms race between plants and their attackers is to identify effector proteins released by the attacker during the interaction with the plant. This has been extensively done for microbial plant pathogens. Subsequently, the identified effectors are either used to directly identify resistance genes, usually in wild germplasm, or to identify their targets in the plant (Vleeshouwers and Oliver, 2014; Khan et al., 2018). The latter approach can provide valuable information to understand the effector-mediated mechanism of disease and thus explore novel strategies for resistance-breeding programs. Recently it has been shown that aphids and spider mites produce effector proteins in their saliva to manipulate plant immunity (Bos et al., 2010; Mugford et al., 2016; Villarroel et al., 2016). Interestingly, an effector from aphid (*Myzus persicae*) was found to target a protein involved in endomembrane vesicle trafficking in *Nicotiana benthamiana*, demonstrating that herbivorous insects utilize effectors to target host proteins and to increase their virulence on the host (Rodriguez et al., 2017).

The recent banning of key chemicals used for controlling thrips infestations and the emergence of heritable pesticide resistance in thrips (Kirk and Terry, 2003) has emphasized the importance of identifying genetic plant resistance against thrips. However, the toolbox for studying genetic resistance to this insect is still very limited. The main reason for this is that studying the contribution of individual genetic factors in crop plants often require the generation of stable transgenic lines. Moreover, the small size and fast movement of thrips, especially adults, make them champions in escaping from experimental bioassay setups and hence the results of experiments may suffer from bias. Although screens which rely on transient gene expression in model plants has been used to identify effectors from other herbivorous arthropods (Bos et al., 2010; Villarroel et al., 2016), the application of this methodology for thrips might face a number of challenges. First, the use of model plants, although ensuring efficient gene expression, might be suboptimal as host and thus less suitable for evaluating insect performance. As a consequence, this can lead to a failure in correctly measuring the response caused by the treatment and may cause inconsistencies during verification in host plants. Second, suitable hosts are often not optimal for transient gene expression by the traditional pressure-based agro-infiltration of leaves. This is either due to the low transformation competence or the induction of necrosis, like in tomato leaves when pressure-infiltrated with the *Agrobacterium tumefaciens* strain GV3101 (Wroblewski et al., 2005). In that case, necrosis is visible at the infiltration sites in response to the used bacterial strain and the wounding caused by the infiltration. It is therefore often difficult to obtain a uniform expression in the host tissue which leads to an increase in treatment-independent variations in the final bioassay results. To overcome these issues, we have optimized a functional analysis method to screen for genes required for thrips reproductive performance in large tomato leaf discs. We demonstrate the feasibility of this approach by the transient ectopic expression of a set of effector candidate-genes from thrips and then evaluating thrips reproductive performance. The expression of enhanced GFP (eGFP) is used as negative control to which thrips reproductive performance in the treatments is compared with and to simultaneously monitor the level of GFP expression throughout the experiment. This protocol provides several advantages among which: (a) it allows for necrosis-free, transient expression in tomato leaf tissue due to the use of *A. tumefaciens* strain 1D1249 (Wroblewski et al., 2005); (b) it establishes protein expression for as long as 12 days post-infiltration with peak expression at day 5. This time frame exceeds the total time required for the screen including the development of nymphs which is 8 days; (c) it increases the homogeneity of protein expression in the tissue due to efficient vacuum infiltration of *A. tumefaciens* followed by a recovery-from-infiltration procedure; (d) it provides efficient containment of adult thrips and their progeny during the course of the experiment which eliminates the need for a greenhouse equipped to accommodate the combination of agro-infiltrations and thrips infestations; (e) it is conducted in 6-well plates, thus requiring a relatively small space, and is therefore conducted under controlled conditions in a climate box which increases reproducibility between experiments; (f) due to compactness of the setup, one can screen multiple genes with enough replicates for statistical analysis; (g) different plant species can be used for screening either effectors or plant genes to identify their significance to thrips reproductive performance and, finally, (h) the method can be used for screens with other small insect pests or microbes.

## Materials and Methods

### Isolation and Cloning of Thrips Effector Candidate-Genes

RNA was extracted from *F. occidentalis* samples, anesthetized by incubation on ice for 1 min then flash frozen in liquid nitrogen, stored in liquid nitrogen, according to the Trizol procedure (TRI Reagent, T9424, Sigma) followed by Turbo DNAse treatment (TURBO DNAse, AM1907, Thermo Scientific) and first strand cDNA synthesis using 5 μg RNA and the RevertAid H Minus Reverse Transcriptase (Thermo Scientific, K1631). Initial thrips candidate effectors were selected by proteome prediction from cDNA sequences that we have generated from sequences obtained from the public Sequence Read Archives (SRA) for thrips whole body and salivary gland transcript sequences (SRX457725, SRR1826954, SRR1826955 and SRR1826956). We selected cDNAs that encode relatively small proteins (50–300 amino acid residues), predicted to have signal peptides by SignalP4.1 (Petersen et al., 2011), lack transmembrane domains (using TM-HMM, DTU Bioinformatics) and predicted to be secreted outside the cell by WolfPSort (Horton et al., 2007). Primers were designed to contain the Gateway (Thermo Scientific) attB sites fused to ATG at the start codon and the cDNA-specific sequence encoding the mature protein. Similarly, the gene encoding eGFP was amplified and the generated expression construct used as a negative control during the screen. PCR amplification from the generated cDNA was conducted using Phusion polymerase (Thermo Scientific, F530S) according to the manufacturers' protocol and then directly purified from reaction (Thermo Scientific, K0702). BP reactions were conducted using pDONR221 as recipient vector with BP clonase and the reactions were transformed to electro-competent *Escherichia coli* DH5α cells. Positive colonies were selected on growth medium supplemented with 50 μg/ml kanamycin and verified by colony-based PCR using the plasmid M13 forward primer and a gene-specific reverse primer. Plasmids were isolated (Thermo Scientific, K0503) from two independent clones and the insert was confirmed by sequencing (Macrogen). Verified entry plasmids were used for LR reaction with either the *in planta* expression vectors pGWB512 (Nakagawa et al., 2007) for expression in tomato or pTRBO-FLAG-GW (Figure S2) for expression in *N. benthamiana*. pTRBO-FLAG-GW was modified from Lindbo (2007) by incorporating the FLAG peptide (Hopp et al., 1988) and the Gateway cassette, containing the R1 and R2 recombination sequences, at the PacI and NotI restriction sites and verified by sequencing. Both vectors add an N-terminal FLAG tag to the mature protein. The LR reaction products were transformed to *E. coli* DH5α cells and positive clones, growing on 30 μg/ml spectinomycin, were selected and verified by colony-based PCR using a plasmid forward primer and a gene-specific reverse primer. Subsequently, plasmids were isolated and used to transform electro-competent *A. tumefaciens* strain 1D1249 for expression in tomato (Wroblewski et al., 2005).

### Thrips Rearing

**Figure 1:**
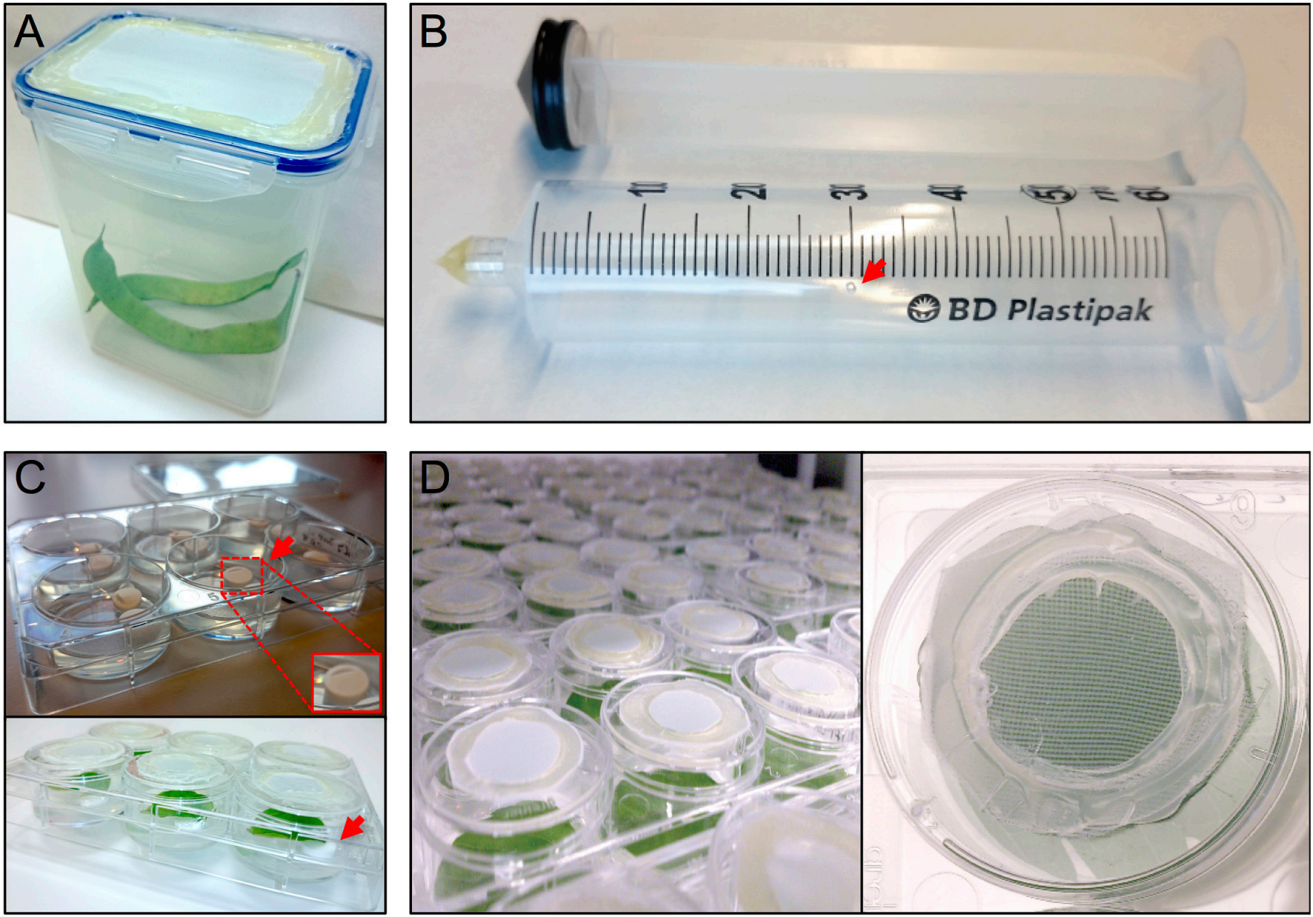
Setup thrips rearing, vacuum infiltration and plate assay. **(A)** thrips rearing box with an open lid that has been sealed with mesh, **(B)** a 50 ml syringe converted to a vacuum infiltration device with a ventilation hole (arrow), **(C)** leaf discs are lifted away from the agar from one side using tube coders (arrows), and **(D)** modified 3.5 cm ø 6-well plate caps with an open top sealed with mesh.

The thrips colony was maintained in 1 L plastic containers with a side opening, or a cover containing an opening, that had been sealed with thrips mesh (SEFAR NITEX, 03-80/37, Figure 1A). Each box was regularly supplemented (after the formation of L2 nymphs) with fresh, cleaned, common bean pods and typha (*Typha latifolia*) pollen. The boxes were maintained in a closed climate box (ECD01, Snijders Labs) at 21°C, 16 h light and 65% RH. Under these conditions, a complete cycle of a developmental-stage synchronized thrips colony, from oviposition to the production of adults, takes 3.5–4 weeks (Note 1). First, ~300 female adult thrips were collected and allowed to lay their eggs for 24 h into the surface of fresh common bean pods, to which typha pollen were added. Next, the adults were removed and the beans were further incubated for 5 days until the emergence of the nymphs, which were fed with pollen twice a week until they developed into adults after 2 more weeks.

### Preparation of *A. tumefaciens* Inoculum

*A. tumefaciens* strains carrying expression constructs for eGFP or genes to be screened were inoculated from overnight pre-cultures in a shaking incubator (28°C and 225 rpm, Innova 4330, Brunswick Scientific) so that the optical density (O.D._600_, OD), obtained the next day when harvesting the cells, is between 0.8 and 2.0 (Note 2). This can be done by adding X microl of a pre-culture that was grown overnight to 10 ml of sterile YEP medium (Bacto-Trypton, 10 g/L; yeast extract, 10 g/ L; NaCl, 5 g/ L; pH 7.5) in 50 ml sterile tubes and incubating at 28°C and 225 rpm (Note 3), where X = (Z/OD)/10), Z = 8,0000/(2^∧^ (ΔT)/2), and ΔT = the time between medium inoculation and harvesting the cells (Note 4). On the day of infiltration, the OD of the bacterial cultures was determined and cells were harvested by centrifugation at 3,600 g and 21°C (Hettich Rotina 420R, Sigma) after which supernatant was discarded and each pellet was resuspended in a volume of infiltration medium (20 g/L sucrose, 5 g/L MS basal salt mixture without vitamins, 1.95 g/L MES, pH = 5.6 and 1 ml/L 200 mM acetosyringone) to reach the final infiltration OD = 0.3 for each treatment. To vacuum-infiltrate 12 leaf discs, ~30 ml of this infiltration-ready culture is required.

### Plant Material and Preparation of Leaf Discs

On the day of the vacuum infiltration with *A. tumefaciens*, leaf discs, 3 cm ø, were prepared using a cork borer from the third and fourth, fully stretched, true leaves of 3.5 to 4-week-old tomato (Moneymaker, growing in 15 cm ø pots at 21°C, 16 h light and 65% relative humidity) or 4-week-old *N. benthamiana* plants by cutting leaflets, excluding the older terminal leaflets, on thick Whatman paper using a sharpened cork borer. The leaf discs were collected into a 1 L beaker filled with tap water to wash the leaf discs and remove cell debris at the leaf disc circumference. Once all leaf discs have been prepared, the water was refreshed twice to wash and reduce damage components. Just before the vacuum infiltration with *A. tumefaciens*, the leaf discs were surface dried on a clean tissue and placed as two stacks of 6 leaf discs in preparation for vacuum infiltration.

### Infiltration and Recovery of Plant Leaf Discs

Per treatment, a 50 ml syringe (BD Plastipak, 300865) was converted into a vacuum infiltration device by either melting or blocking the syringe tip using hot glue (11 mm ø, Mascot Europe BV) and making a small ventilation hole (1 mm ø) in the syringe barrel at the 30 ml mark (Figure 1B).

Two groups of the previously prepared leaf discs (6 stacked leaf discs per group) were gently placed into the syringe barrel, after removing the syringe plunger, one after another and by gently striking against a hard surface until they almost reach the bottom (Note 5). Subsequently, the barrel was filled with the infiltration culture until just below the ventilation hole. At that point, the leaf discs should be submerged in the bacterial culture. The plunger is placed back into the barrel in such a way that air, which is trapped above the surface of the culture, is allowed to escape via the ventilation hole. After air removal, the ventilation hole was closed using a gloved finger and the plunger was simultaneously pulled out from the barrel to generate a negative pressure. At that point air bubbles were formed, indicative for the extraction of air trapped in the intercellular space of the plant tissue. The extracted air was allowed to leave the liquid and reach the seal of the plunger by holding the syringe vertically and gently striking two times against a hard surface. Releasing the finger from the ventilation hole or, alternatively, pushing the plunger back into the barrel while keeping the ventilation hole closed neutralized the negative pressure and caused the infiltration of the leaf tissue after which the leaf discs appeared as being water soaked. Subsequently, the culture was discarded and the leaf discs were removed from the barrel by striking the barrel opening against a thick filter paper positioned on a hard surface. The surface of the leaf discs was dried using clean tissue and the leaf discs were placed, with abaxial side pointing upwards, on a shallow cover of a large Petri dish (Greiner, 145 mm, Z652539) containing wet sterilized chromatography paper (Whatman, 3MM CHR, 3030-917) and allowed to recover from the infiltration in a down flow cabinet for 45 min and up to 1 h (Note 6).

After recovery, each leaf disc was positioned with adaxial side up in a single well of a 6-well plate (Greiner, 657160) containing 4 ml of 0.75% sterile water agar (Daishin agar, Duchefa, D1004.1000), prepared using ultrapure water (MQ, Milli-Q System, Millipore Corporation). Leaf discs were positioned in their wells so that the thick side of the midrib (previously the closest to the leaf base) is in contact with the agar. The opposite side of the midrib was resting on two surface sterilized lifters (Figure 1C) (Nalgene cryogenic vial coders, Thermo Scientific, 5045-0000) (Note 7). After loading a plate with leaf discs, their contact points with the agar surface were pressed slightly into the agar using tweezers to ensure sufficient but shallow contact (Note 8). Subsequently, the wells were closed using individual lids (Thermo Scientific, Nunc, 150350) equipped with thrips mesh to prevent thrips from escaping the wells and allow the exchange of air and humidity during incubation inside the climate box (ECD01, Snijders Labs, Figure 1D). For this, each lid was modified by making a 2 cm ø hole at the center and then sealing the created opening with thrips mesh (Figure 1D) using hot glue. Subsequently, leaf discs were allowed to further recover in the climate box for 1 day. At this point, the plates were not covered with the original rectangular plate-cover in order to allow a better ventilation and thus facilitate the leaf disc recovery.

### Thrips Starvation

**Figure 2:**
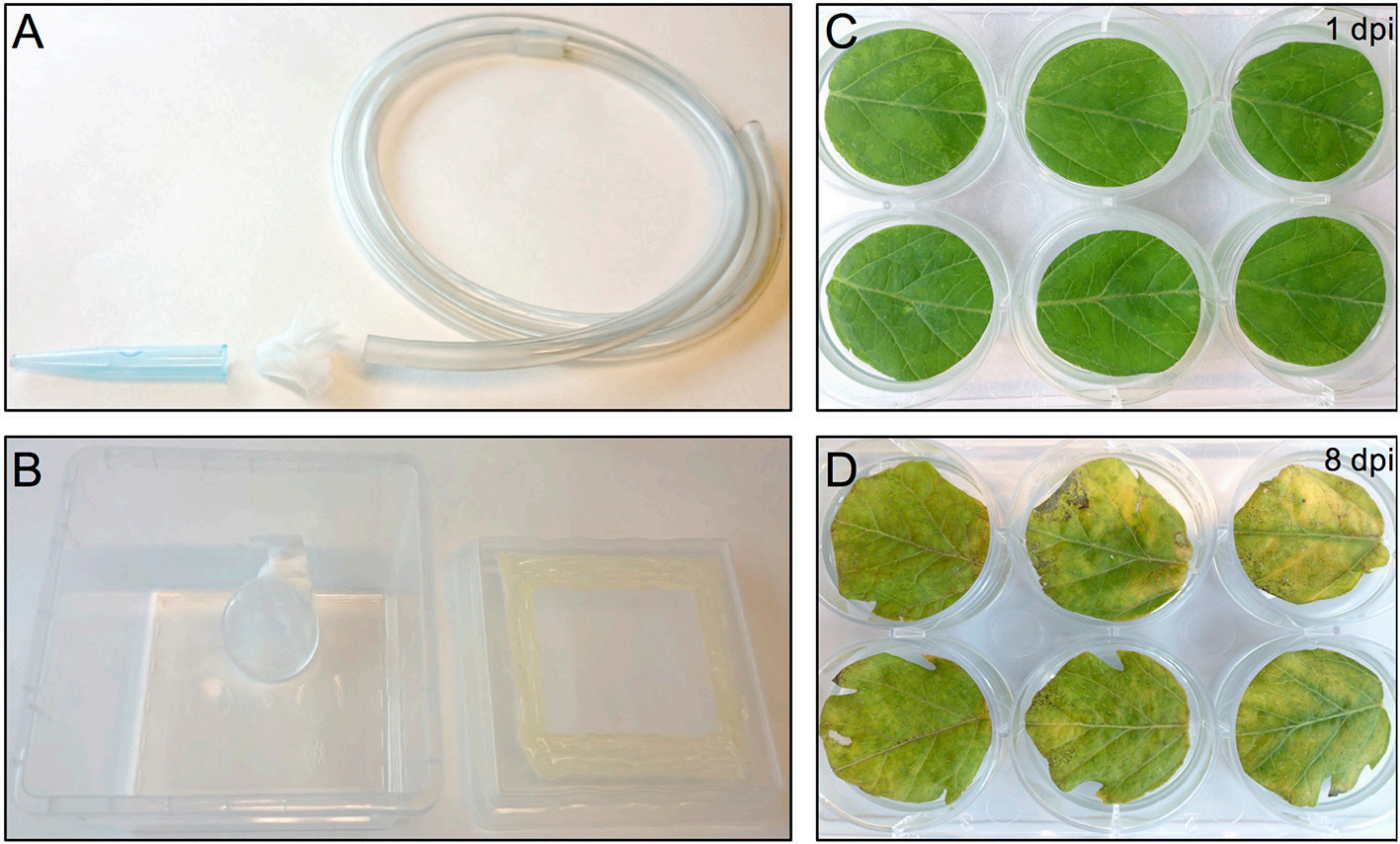
Thrips collection and starvation setup and the effect of agro-infiltration on leaf-disc phenotype. **(A)** Small size plastic hose covered at one end with mesh and a 1 ml pipette tip used to capture thrips by sucking. **(B)** Thrips starvation setup consisting of a plastic box in which a small water container has been placed, covered with a stretched layer of Parafilm. The lid of the box contains an opening that has been covered with mesh. **(C)** Tomato leaf discs infiltrated with *A. tumefaciens* strain 1D1249 carrying an *in planta* expression construct at 1 dpi or at 8 dpi **(D)**.

One day before infesting the plant leaf discs with thrips, adult age-synchronized female thrips were collected from the rearing boxes, excluding dead thrips and pollen, by sucking individual adults into a 50 ml disposable tube. Adults were collected from the original rearing box by mouth-aid air suction, using a transparent, small size plastic hose covered at one end with a filter consisting of a small piece of mesh and jammed into a 1 ml pipette tip of which the end has been cut off to create a larger opening (Figure 2A) (Note 9). Although 5 female adult thrips are required for each well, ~40% more were collected to compensate for loss during starvation and contamination with male adult thrips. Subsequently, a second round of cleaning was applied by releasing the collected adult thrips into the center of a large clean shallow box and collecting adults that migrate away from the center. Water was offered to the double-cleaned adult thrips in a water container which is made by filling a cover of small cell culture plate (Thermo Scientific, Nunc, 150350) with tap water and covering the top with a stretched layer of Parafilm (Parafilm M, Sigma, P7793-1EA) (Note 10). The water plate was fixed by pressing the excess of the Parafilm to the side of a plastic box so that it is resting on the bottom of the box at a 45° angle which allows air to go to the top and thus facilitate the utilization of water via the Parafilm by thrips (Figure 2B). Finally, the box was covered with mesh and starvation was sustained for 24 h in the climate box.

### Thrips Bioassay

After 1 day recovery of the leaf discs in the climatebox, for each well 5 starved female adult thrips were collected into 1.5 ml microtubes. Once all tubes were filled, they were randomized and placed in groups of 12, sufficient for infesting 2 plates (12 leaf discs) for each treatment. Infestation and oviposition were started by removing the adjusted lids from the wells of two plates, each containing 6 recovered leaf discs, and placing the two plates and 12 thrips tubes on ice for 1 min (Note 11). Directly after, the adults were added to each plate by tapping each tube content in each well and checking if all 5 adults have been transferred to the well. This process was conducted quickly to avoid thrips from gaining consciousness. Once adults were added to all wells in one plate, the wells were quickly closed with the adjusted individual lids. The same was done for the second plate. Next, the two plates were closed with the original rectangular plate-cover and transferred to the climate box for a 24 h infestation.

After infestation, adult thrips were scored per well (dead/alive) for each treatment before being removed. The thrips-free wells were closed with both the adjusted individual lids and plate cover and further incubated in the climate box for 5 days. On day 8 post-infiltration, the nymphs in each well were counted using a stereo microscope (M3, Wild-Heerbrugg).

### Statistical Analysis

A single measure parametric analysis was conducted using InVivoStat (Bate and Clark, 2014) on the data from each screen using a one-way ANOVA analysis. When significant differences with the negative control are encountered, the analysis is followed by comparisons of the predicted means of the treatment factor (the over-expressed gene) back to the control (eGFP) group mean using the Least Significant Difference (LSD) procedure.

### GUS Assay

Leaf discs were agro-infiltrated as described above using *A. tumefaciens* strain 1D1249 carrying the plasmid pGWB512-GUS and GUS activity was measured at different time points post-agro-infiltration. For each time point, three leaf discs were vacuum-infiltrated in 40 ml GUS buffer supplemented with X-gluc (5-Bromo-4-chloro-1*H*-indol-3-yl β-D-glucopyranosiduronic acid, Thermo Scientific, R0851), using a modified syringe as the one used for agro-infiltration. First 500 ml GUS buffer (50 ml of 1 M phosphate buffer, pH 7.5, 5 ml Triton X-100, 5 ml DMSO, 10 ml of 0.5 M EDTA, pH 8 plus 635 ml demineralized water) were prepared without the addition of the GUS substrate and stored at 4°C. Directly before infiltration, 40 ml of the GUS buffer were allowed to reach room temperature and then 200 μl of the X-gluc stock (100 mM in DMSO) were added and mixed thoroughly. After vacuum infiltration, leaf discs and the infiltration buffer were incubated at 37°C for 24 h. The blue product that is formed due to the cleavage of the substrate X-gluc by GUS activity is diffused into the buffer, as tissue fixation was omitted, and subsequently measured per ml buffer/leaf disc (~100 mg tissue) by absorbance at 460 nm. The obtained values reflect the difference in GUS activity and thus the level of *GUS* gene expression among the samples. A list of all used equipment is provided in Supplementary Material.

### Notes

1. The use of a synchronized thrips colony makes it easier to distinguish female adult thrips from male adult thrips, as the latter are slightly smaller in size, and thus reduces the variations in nymph counts among treatments.
2. It is important to maintain this OD to ensure that the cells are in the logarithmic growth phase and thus reduce variability between treatments and increase reproducibility.
3. *A*. *tumefaciens* strain 1D1249 grown as a liquid culture can form aggregates. We found that growing this culture at 28°C in a shaker set to a value between 225 and 250 rpm reduced aggregate formation without affecting the final expression levels after agro-infiltration.
4. If the OD on the day of infiltration is still too low then the value of ΔT in the formula can be reduced with 1 or 2 h without adjusting the actual number of hours used for the incubation. Similarly, if the OD appears to be higher than desired on the day of infiltration, the value of ΔT can be increased with 1 or 2 h.
5. It is important to avoid causing any damage to the leaf discs during the whole procedure to avoid triggering wound responses. This is also why the leaf discs should be cut using a cork borer with a sharp edge to minimize tissue damage.
6. The cover of a Petri dish is used as it is shallow and when placed near to the air stream in the down flow, it allows air to circulate around the leaf discs and thus improve water evaporation from the intracellular space. This rapid recovery step is meant to evaporate the excess of water and remove the water soaking phenotype from the infiltrated leaf discs in a short time but without allowing the them to dry out. The recovery from infiltration is necessary to prevent hypoxia which can otherwise lead to tissue necrosis. Although the leaf discs are not sterile, we use clean or sterilized material to reduce the chance of developing fungal infection on the leaf discs during the course of the experiment.
7. The use of the lifters allows further recovery of the leaf discs from infiltration during incubation, maintains better humidity around the leaf disc and allow the adult thrips and the nymphs to move freely around the leaf disc. The cavity of the lifters should be facing the agar surface. This prevents trapping humidity if they were facing the leaf tissue which increases the chance of fungal infection.
8. A shallow contact with the agar surface is important to reduce tissue necrosis at this region of the leaf disc due to hypoxia.
9. It is important to avoid collecting dead thrips to which pollen are usually stuck or collect the pollen themselves which will disturb the starvation process.
10. Avoid stretching the Parafilm more than one time its length to prevent possible water leakage and drowning the thrips.
11. Depending on the temperature of the surrounding, the incubation time on ice for thrips can be decreased as long it will remain anesthetized. Do not exceed the 1 min as it can lead to mortality.

## Results

### Vacuum Infiltration and Leaf Disc Recovery

**Figure 3:**
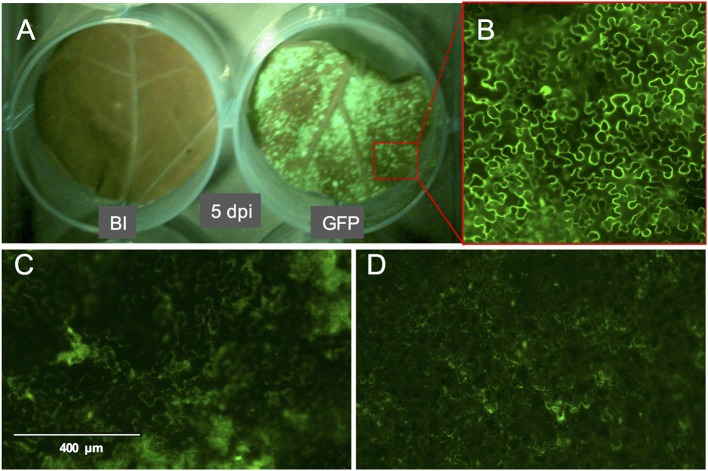
GFP fluorescence signal on whole leaf discs of *Nicotiana benthamiana* at 5 dpi and the phenotype of the GFP signal. **(A)** *N. benthamiana* leaf disc infiltrated with buffer (left) or with *A. tumefaciens* strain GV3101 carrying pTRBO-FLAG-GW (right) at 5 dpi. **(B)** Microscopic image of GFP signal from the leaf disc in **(A)**. **(C,D)** GFP signal phenotype observed as a result of hypoxia when the leaf discs were not sufficiently recovered from the vacuum infiltration of *A. tumefaciens* in *N. benthamiana* **(C)** or tomato **(D)**.

Compared to pressure infiltration or the infiltration using a conventional vacuum pomp, our infiltration procedure allowed us to obtain complete tissue infiltration where the water soaking phenotype was observed on the whole tissue. This is despite that we have used large leaf discs which are usually more difficult to infiltrate. Also, the leaf discs did not suffer from wounding as that caused by pressure infiltration. We found that tomato leaf discs of the cultivar Moneymaker recover slightly faster (45 min to 1 h after infiltration) from the water-soaking phenotype, caused by the infiltration, than *N. benthamiana* leaf discs (1 h after infiltration). We found it to be essential that the infiltrated leaf discs reach a near-complete recovery before being transferred to the agar plates, otherwise tissue necrosis could appear on them 2–3 days later and then they become more vulnerable to fungal infection. Although protein expression was often still visible in these non-recovered regions, it was somewhat diffused as observed after the expression of eGFP (Figures 3C,D). Notably, the recovery of the leaf discs from the infiltration needed to be continued on agar. Immersing a large part of the leaf disc into the agar or having direct contact of the complete leaf disc surface with the agar often resulted in reoccurrence of the water soaking phenotype and necrosis often developed at these contact regions. To solve this, the leaf discs were lifted from the agar from one side using clean plastic micro-tube coders (see materials) while the thick side of the midrib remained slightly inserted into the agar. Failing to use these settings caused a prolonged presence of water soaked tissue and the development of necrosis at the positions where the leaf disc was contacting the agar. Also, thrips benefit from this as adults, and later, nymphs are often encountered at the abaxial side of the leaf disc which becomes more accessible due to lifting the leaf discs.

### Protein Expression

**Figure 4:**
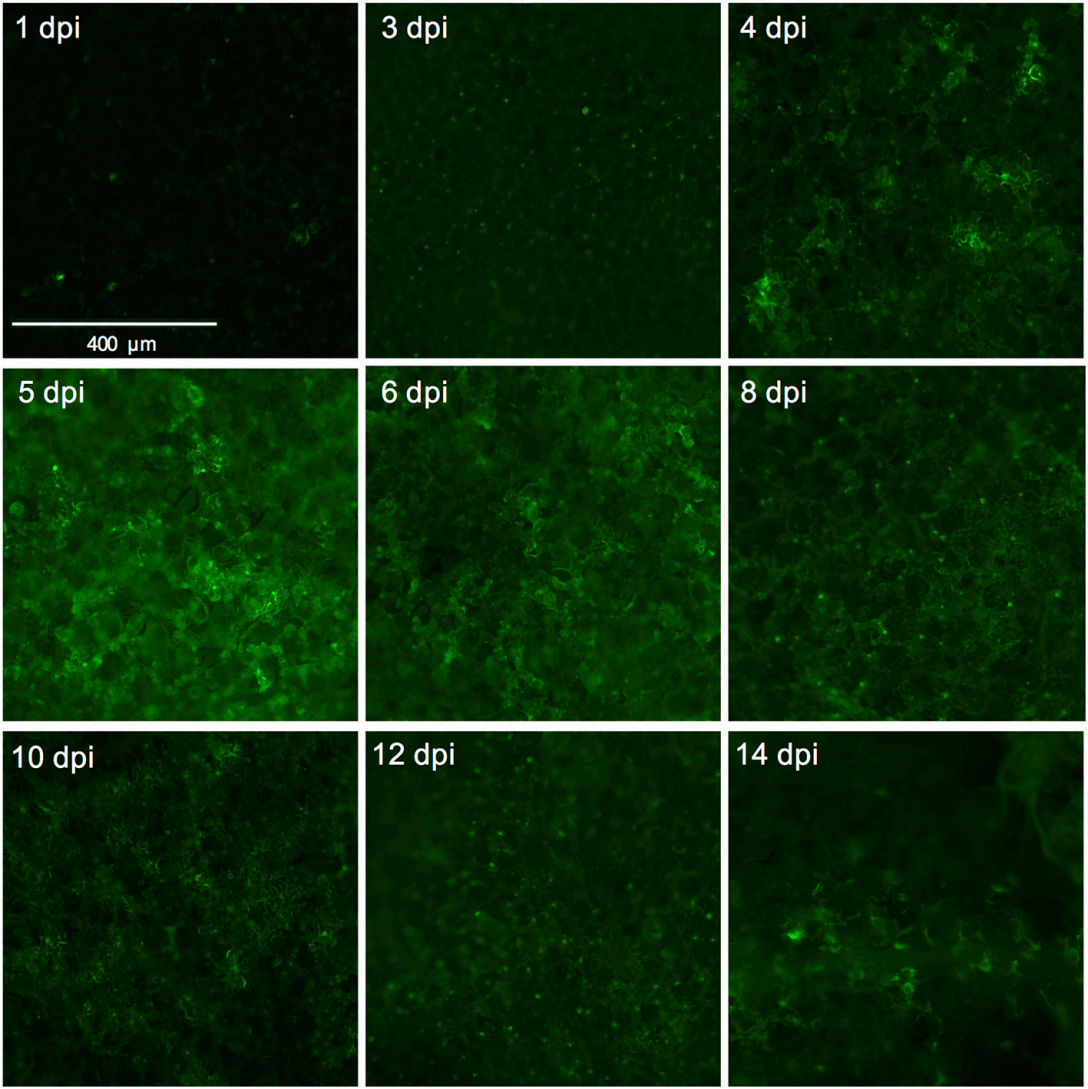
Successful expression of GFP in tomato leaf discs after vacuum infiltration with *Agrobacterium tumefaciens* and recovery from infiltration. Leaf discs prepared from 3.5 to 4-week-old tomato plants of the cultivar Moneymaker were vacuum-infiltrated with the *Agrobacterium tumefaciens* strain 1D1249 carrying the plasmid pGWB512 in which the eGFP is expressed under the control of the 35S promoter of cauliflower mosaic virus. Leaf discs were allowed to recover from the infiltration for 1 h under an air stream and then placed on water agar in 6-well plates. Pictures were taken using a wide-field fluorescence microscope at the indicated time points and magnification.

We used the eGFP control as an indicator for protein expression during the course of the experiment by monitoring GFP fluorescence. The GFP signal was visible at 4 dpi and continued to increase at 5- and 6 dpi after which it started to decline at 8 dpi. At 14 dpi the signal was much weaker and was often confined to the areas adjacent to the veins (Figures 3A,B, 4).

### Containment of Thrips and Bioassay Optimization

Counting the number of the adult female thrips after infestation and oviposition showed that no escapes occurred, which reflected efficient containment of thrips in the utilized experimental setup. Moreover, a low mortality rate (1–2%) was observed which indicates that adult thrips can cope with the defenses existing on the treated tomato leaf discs. This is different from the mortality rate that we observed in treated *N. benthamiana* leaf discs and that was up to 35% (data not shown), indicating the high toxicity of that species for thrips.

After several trials, we found that it is important to optimize the number of adult female thrips used for infestation and oviposition, the contact time with the plant leaf discs and the incubation time of the leaf discs before counting the nymphs. This was particularly important to reduce sample to sample variation and increase the reproducibility of the results. Accordingly, we found that it is optimal to use 5–6 female adults per leaf disc (3 cm ø). This ensures obtaining a reasonable number of nymphs that can be counted concurrently in 1 day for a single screen containing 15 treatments, including the controls. The infestation and oviposition was allowed for 24 h in the climate box. We found this to be important for generating a synchronized emergence of the progeny so that most eggs would have hatched and could be counted in the scoring day. Under these experimental settings, the optimum incubation time that allowed most eggs to hatch was between 7 (in the summer) and 8 (in the winter) days post-oviposition. This incubation time might differ slightly depending on the tested plant species as thrips reproductive performance is known to vary among plant species (Baez et al., 2011).

### Identifying Genes That Influence Thrips Reproductive Performance

**Figure 5:**
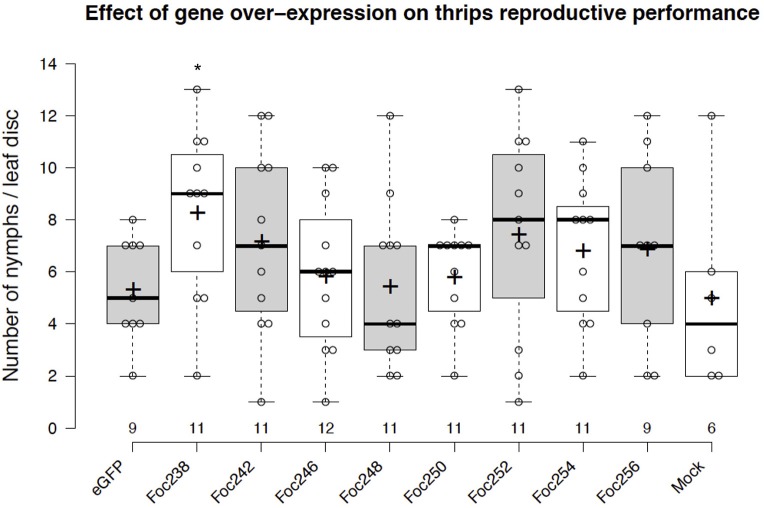
Effect of transient expression of thrips effector-candidate genes on thrips reproductive performance. The box plot represents the results from an effector screen, showing the number of nymphs scored at 8 days post-agro-infiltration to transiently express eGFP (control) or thrips effector candidate genes in tomato leaf discs. Oviposition by adult female thrips was started at 2 dpi and continued for 24 h. Mock represents leaf discs infiltrated with only infiltration medium. The box center-lines represent the medians; box limits indicate the 25th and 75th percentiles as determined by R software; whiskers extend 1.5 times the interquartile range from the 25th and 75th percentiles; outliers are represented by detached dots; crosses represent sample means; data points are plotted as open circles; n is between 6 and 12 leaf discs. ^*^LSD, *P* = 0.07.

The plotted nymph counts showed variations within the treatments, but also between some treatments and the negative control (eGFP). None of the thrips effector-candidates that were tested here showed statically significant difference at the 5% level on nymph counts, compared with the control (Figure 5). However, expression of the effector candidate Foc238 led to a near significant (*P* = 0.07) enhancement of thrips reproductive performance as the number of nymphs was higher than in the negative control. Thus, Foc238 is a putative effector that can now be further tested with increased sample sizes. Foc238 is predicted to contain a complete open reading frame, which encodes a small protein (163 AA) with a signal peptide and two repeat domains. It is highly expressed in the salivary glands of thrips and has no significant similarity with known sequences from other organisms. We are currently using this screening method to study a large set of putative effector candidates from thrips in a medium throughput setup to find those that have a stronger effect on the reproductive performance of the thrips.

## Discussion

The tremendous increase in sequence-based genetic information from plants and their herbivores and the quest for plant genetic resistance requires rapid genetic screening procedures to identify resistance-related traits. Our screening procedure addresses this by eliminating the need for generating stable transgenic plants and to be able to screen with herbivorous insects for up to, at least, 8 days. It also allows conducting the screen under strictly controlled conditions and in a relatively small space which increases the reproducibility of the results. Another benefit is the cost reduction due to omitting the occupation of a large greenhouse space equipped to accommodate the combination of genetic screens with genetically modified organisms and insect bioassays. Furthermore, the procedure allows a necrosis-free, transient protein expression in tomato leaf tissue. This is accomplished by the use of *A. tumefaciens* strain 1D1249 in a setup that results in prolonging the duration of protein expression to encompass the time required for finalizing the thrips bioassay. Although infiltration of the tomato leaf discs with the *A. tumefaciens* strain 1D1249 does not induce necrosis on the leaf discs, we observed chlorosis which was especially most pronounced at the end of the screen, starting from day 8 (Figures 2C,D). The observed chlorosis was similar to that formed when infiltrating leaves attached to whole plants with this strain (data not shown). However, this did not prevent protein expression until the end of the thrips bioassay as observed in the eGFP control treatments (Figure 4) and on the whole leaf-disc level after expression of the *GUS* gene (Figure S1).

The utilized vacuum infiltration method, followed by a recovery-from-infiltration procedure, increases the homogeneity of protein expression in the tissue. This is important to reduce the score variations among samples of the same treatment and increases the reproducibility of the results. The use of an age-synchronized thrips colony and the efficient containment of adult thrips and their progeny during the course of the experiment also contributes to reducing variations. It is possible to use this method for other plant species to screen either single effectors or plant genes or to screen wild accessions to identify their significance to thrips reproductive performance. In the latter, the screen becomes even faster as the vacuum infiltration and recovery steps are not required. Similarly, rapid genetic functional analysis techniques like virus-induced gene silencing can also be combined with this screen, where silenced plants are used to generate leaf discs that are directly used without the need for agro-infiltration and recovery. Despite that one of the aims of this procedure is to reduce the within-treatment variations, we still see them. This may have to do with the complexity of the involved biological system which includes plant, bacteria and thrips. Therefore, treatments that cause small effect on thrips performance might not directly show statistically significant difference. A possible solution for this could be to increase the sample size, although this will limit the number of genes that can be tested in one screen. Thrips effectors that have positive or negative effect on thrips reproductive performance are very useful to include in the screen. However, there are no thrips effectors known to date. Instead we expressed several known aphid effectors (Mp10, MpC002, and Mp42) (Bos et al., 2010) and we also cloned and tested a thrips homolog of one of these aphid effectors, Mp10 (Mugford et al., 2016) and evaluated thrips performance. However, the expression of none of these genes affected thrips reproductive performance in our assay.

Finally, this method can be used to identify genes that play a role in the plant interaction with other small pests or microbes as long as the screen can be conducted within the time frame of expression. However, we think that in the future there is room for further optimization of this method by using more potent expression vectors and *A. tumefaciens* strains selected for high transformation efficiency, especially on the studied plant species. The desired improvements would be extending the duration of maintaining plant tissue in a healthier state while obtaining sufficient levels of protein expression. This will allow using this approach for studying other organisms for which the screen requires a longer time frame.

## Author Contributions

AA designed the experiments. AA and SH conducted the experiments. AA, SH, and RS edited the manuscript.

### Conflict of Interest Statement

The authors declare that the research was conducted in the absence of any commercial or financial relationships that could be construed as a potential conflict of interest.
