# Supplementary material for: A Robust Functional Genomics Approach to Identify Effector Genes Required for Thrips (Frankliniella occidentalis) Reproductive Performance on Tomato Leaf Discs
Source: Front Plant Sci. 2018 Dec 13;9:1852. doi: 10.3389/fpls.2018.01852 (PMC6301195; doi:10.3389/fpls.2018.01852)
Supplement: Supplementary file 1 [file Data_Sheet_1.docx]

**SUPPLEMENTARY MATERIAL**

**
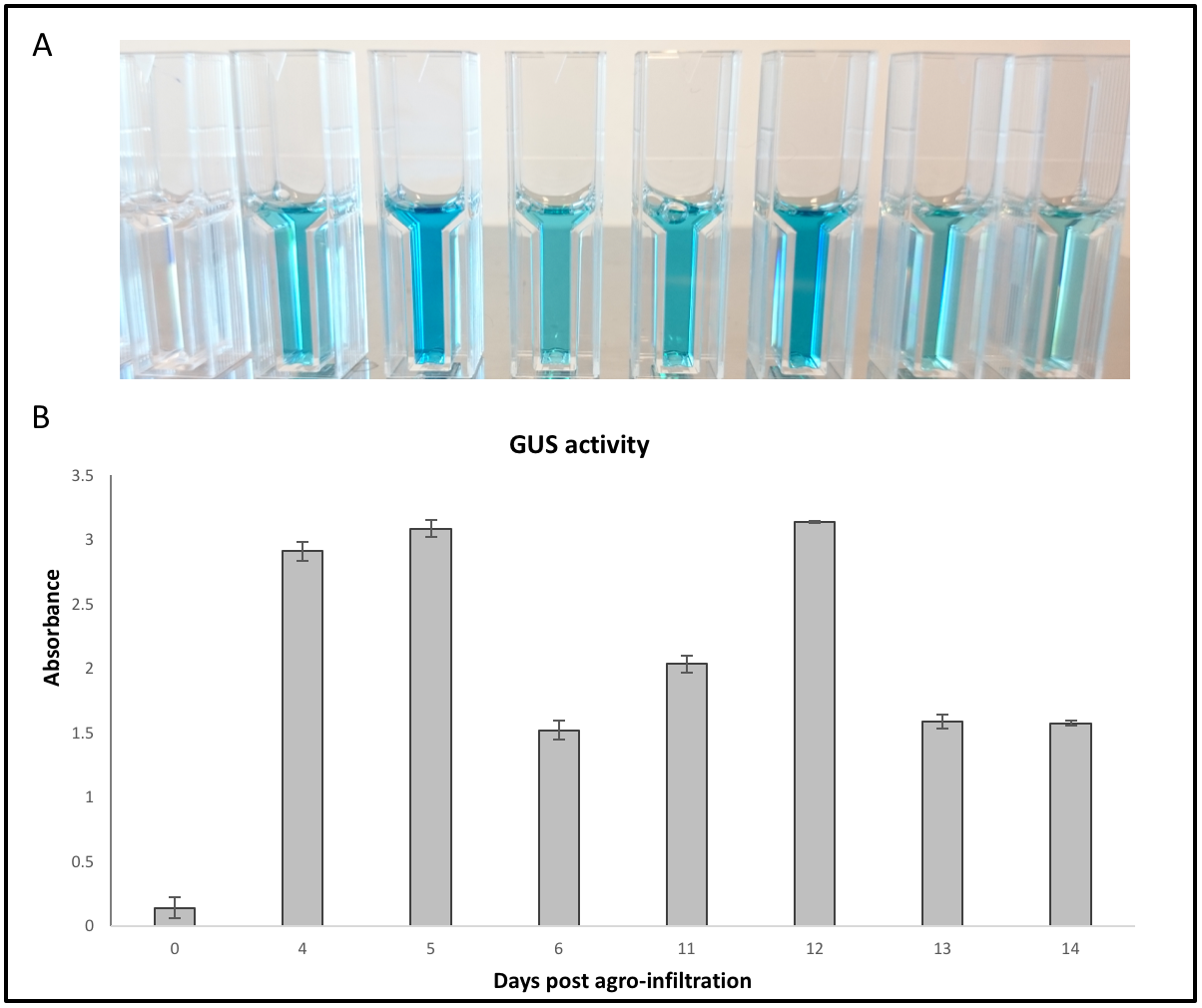
**

**Figure S1: GUS activity after agro-infiltration to transiently express the GUS gene in**

**tomato leaf discs.** Semi-quantitative GUS assay showing (A) the conversion of the colorless

reaction buffer into blue color with different intensities, indicative for differences in GUS activity and the formation of a blue precipitate of chloro-bromoindigo which leaked from the leaf discs into the reaction buffer. (B) Photometric quantification of the blue color intensity, per ml buffer per leaf disc (~ 100 mg), by measuring absorbance at 460 nm. n = 3 leaf discs for each time point.


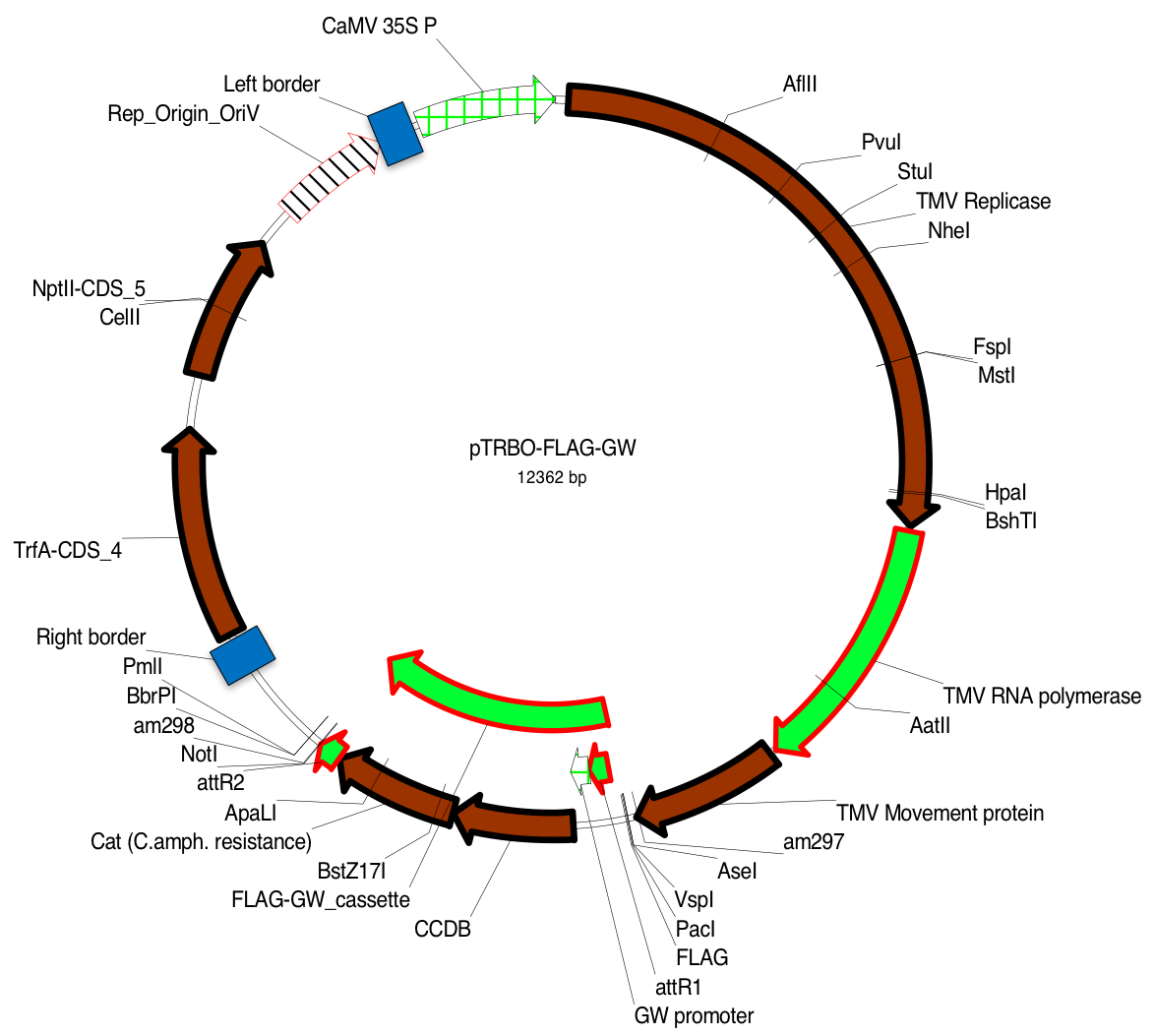


**Figure S2: Restriction & features map for the plasmid pTRBO-FLAG-GW**

pTRBO-FLAG-GW is a modified pTRBO plasmid in which the FLAG peptide and the Gateway cassette, containing the R1 and R2 recombination sequences, were inserted at the PacI and NotI restriction sites.
