# Supplementary material for: A Robust Functional Genomics Approach to Identify Effector Genes Required for Thrips (Frankliniella occidentalis) Reproductive Performance on Tomato Leaf Discs
Source: Front Plant Sci. 2018 Dec 13;9:1852. doi: 10.3389/fpls.2018.01852 (PMC6301195; doi:10.3389/fpls.2018.01852)
Supplement: Supplementary file 2 [file Data_Sheet_2.docx]

**List of Equipment**

1. 3,5 to 4-week-old tomato plants (3 plants / treatment) of the cultivar Moneymaker, growing in 15 cm ø pots containing soil at 21°C, 16 hours light and 65% relative humidity (RH).
2. Cork borer 3 cm ø with sharpened edge.
3. 1 L beaker with tap water to immediately collect the prepared leaf discs.
4. Clean deposable tissue for drying leaf discs before and after infiltration.
5. Tweezers for transferring leaf discs into the 6-well plate.
6. 70 % Ethanol for cleaning between treatments.
7. Thick filter paper, non-sterile (Whatman, 3MM CHR, 3030-917).
8. 6-well plates (Greiner, 657160) containing 4 ml of 0.75% sterilized water-agar (Daishin agar, Duchefa, D1004.1000), prepared using ultrapure water (MQ, Milli-Q System, Millipore Corporation) in each well.
9. Surface sterilized and dried tube coders (Nalgene cryogenic vial coders, Thermo Scientific, 5045-0000), two per well.
10. Thrips mesh (SEFAR NITEX, 03-80/37).
11. Caging-lids (Thermo Scientific, Nunc, 150318). These are used to close the wells in the 6-well plate. Each lid is modified by removing a 2 cm ø circle from the center and sealing that created opening with thrips mesh.
12. Sterile YEP growth medium (Bacto-Trypton, 10 g/L; yeast extract, 10 g/L; NaCl, 5 g/L; pH 7.5).
13. Shaker set to 28°C and 225 rpm (Innova 4330, Brunswick Scientific).
14. 200mM acetosyringone.
15. Freshly prepared minimal infiltration medium with acetosyringone (20 g/L sucrose, 5 g/L MS basal salt mixture without vitamins, 1.95 g/L MES, 1 ml/L 200mM acetosyringone, pH = 5.6).
16. 15 ml and 50 ml disposable tubes.
17. Table centrifuge set to 3600g and 21°C (Hettich Rotina 420R, Sigma).
18. Bacterial cultures for the control (to express eGFP) or the treatments after resuspension in infiltration medium and adjusting O.D._600_ to the required final value for infiltration.
19. One syringe, 50 ml (BD Plastipak, 300865), for each treatment after converting into a vacuum device.
20. Wet sterilized thick chromatography paper (Whatman, 3MM CHR, 3030-917).
21. Climate box (ECD01, Snijders Labs).
22. Lids (Thermo Scientific, Nunc, 150350) and Parafilm (Parafilm M, Sigma, P7793-1EA) for providing thrips with water during starvation.
23. Plastic box with mesh in the lid for thrips starvation.
24. Small size silicon hose covered at one end with mesh and a 1ml pipette tip, of which the end has been cut off to create a larger opening.
25. Starved 3.5 - 4 week old female, mated, adult thrips (5 adults / leaf disc).
26. 1,5 ml micro centrifugation tubes.
27. Icebox with ice.
28. Stereo microscope (Wild-Heerbrugg, M3).
